# The auxin signaling pathway to its PIN transporters: insights based on a meta-analysis of auxin-induced transcriptomes

**DOI:** 10.18699/VJ21.005

**Published:** 2021-02

**Authors:** V.V. Kovrizhnykh, Z.S. Mustafin, Z.Z. Bagautdinova

**Affiliations:** Institute of Cytology and Genetics of Siberian Branch of the Russian Academy of Sciences, Novosibirsk, Russia Novosibirsk State University, Novosibirsk, Russia; Institute of Cytology and Genetics of Siberian Branch of the Russian Academy of Sciences, Novosibirsk, Russia; Institute of Cytology and Genetics of Siberian Branch of the Russian Academy of Sciences, Novosibirsk, Russia

**Keywords:** Arabidopsis thaliana, auxin, PIN-FORMED, auxin-response genes, meta-analysis, gene network, Arabidopsis thaliana, ауксин, PIN-FORMED, ауксин-регулируемые гены, метаанализ полногеномных данных, генные сети

## Abstract

Active polar transport of the plant hormone auxin carried out by its PIN transporters is a key link in the formation and maintenance of auxin distribution, which, in turn, determines plant morphogenesis. The plasticity of auxin distribution is largely realized through the molecular genetic regulation of the expression of its transporters belonging to the PIN-FORMED (PIN) protein family. Regulation of auxin-response genes occurs through the ARF-Aux/ IAA signaling pathway. However, it is not known which ARF-Aux/IAA proteins are involved in the regulation of PIN gene expression by auxin. In Arabidopsis thaliana, the PIN, ARF, and Aux/IAA families contain a larger number of members; their various combinations are possible in realization of the signaling pathway, and this is a challenge for understanding the mechanisms of this process. The use of high-throughput sequencing data on auxin-induced transcriptomes makes it possible to identify candidate genes involved in the regulation of PIN expression. To address this problem, we created an approach for the meta-analysis of auxin-induced transcriptomes, which helped us select genes that change their expression during the auxin response together with PIN1, PIN3, PIN4 and PIN7. Possible regulators of ARF-Aux/ IAA signaling pathway for each of the PINs under study were identif ied, and so were the aspects of their regulatory circuits both common for groups of PIN genes and specif ic for each PIN gene. Reconstruction of gene networks and their analysis predicted possible interactions between genes and served as an additional conf irmation of the pathways obtained in the meta-analysis. The approach developed can be used in the search for gene expression regulators in other genomewide data.

## Abstract (ru)

Активный полярный транспорт гормона растений ауксина, осуществляемый его транспортера- ми, – ключевое звено в формировании и поддержании распределения ауксина, которое, в свою очередь, опре- деляет морфогенез растения. Пластичность распределения ауксина в большой степени реализуется через молекулярно-генетическую регуляцию им экспрессии транспортеров семейства PIN-FORMED (PIN) белков. Ре- гуляция ауксином экспрессии чувствительных к нему генов происходит через ARF-Aux/IAA-зависимый сигналь- ный путь. Однако неизвестно, какие ARF-Aux/IAA белки участвуют в регуляции ауксином экспрессии генов PIN. У Arabidopsis thaliana семейства белков PIN, ARF и Aux/IAA многочисленны, возможны различные комбинации представителей этих семейств в реализации сигнального пути, что создает сложность для понимания механиз- мов этого процесса. Использование данных высокопроизводительного секвенирования транскриптомов, ин- дуцированных ауксином (RNA-Seq), делает возможным обнаружение генов-кандидатов, участвующих в регуля- ции экспрессии белков PIN. Мы разработали алгоритм метаанализа ауксин-индуцированных транскриптомов, с помощью которого отобрали гены, изменяющие свою экспрессию в ответе на ауксин вместе с PIN1, PIN3, PIN4, PIN7, и предсказали возможные регуляторы ARF-Aux/IAA сигнального пути для каждого из дифференциально экспрессирующихся PIN. Применяя сравнительный анализ, мы определили общие и специфичные аспекты в регуляторных контурах, исследуемых PIN. Реконструкция генных сетей и их оценка показали возможные взаимодействия между генами и послужили дополнительным подтверждением большинства сигнальных путей, по- лученных в метаанализе. С помощью комплексного подхода мы предсказали, что регуляция ауксином экспрес- сии PIN происходит через несколько ARF-Aux/IAA регуляторных контуров, опосредованных комбинацией ARF4, ARF10 и IAA4, IAA12, IAA17, IAA18 и IAA32. Часть из них являются специфичными при формировании ауксинового ответа с участием отдельных белков PIN, тогда как другие – общими для нескольких белков PIN. Разработанный алгоритм метаанализа можно применять для решения других задач поиска регуляторов экспрессии генов с при- влечением полногеномных данных.

## Introduction

The key role of auxin in regulation of plant growth and development is a well known fact (Mroue et al., 2018). A significant part of auxin is synthesized in the shoot apical meristems and then transferred to the root, providing there the development of lateral and adventitious roots, as well as the maintenance of the stem cell niche in the root apical meristem. At the cellular level, auxin role in physiological process is carried out by its concentration-dependent effect on cell division and elongation rate (Campanoni, Nick, 2005). Therefore, the formation and maintenance of auxin concentration gradients plays a vital role in morphogenesis. For example, in experiments on root decapitation, it was shown that auxin distribution with a concentration maximum located at a certain distance from the new root tip can be formed again in a few hours (Grieneisen et al., 2007; Mironova et al., 2010). In this case, the regeneration of meristem and normal root functioning occurs only after recovery of auxin distribution pattern (Xu et al., 2006).

The PIN-formed (PIN ) family genes, which encode eight transmembrane transporter proteins in Arabidopsis thaliana, carry out auxin efflux from the cell (Weijers et al., 2001; Petrasek, 2006). PIN1-4, PIN7 transporters are polar localized on the cell plasma membrane, thereby the directed auxin flows are formed in the tissue. For example, at the individual cells level in A. thaliana root tip auxin fluxes forms hormone distribution with maximum in quiescent center (QC), which maintains the stem cell niche in the root (Feraru, Friml, 2008). In most cases, the PIN function is fundamental in formation and maintenance of auxin distribution. It was shown experimentally that there is a complex network of auxin-dependent regulation for PIN expression, which includes positive and negative feedbacks (Gelder et al., 2001; Friml, 2004; Sauer et al., 2006; Vieten et al., 2007). In the article of A. Vieten et al. (2005) it was experimentally shown that treatment with exogenous auxin leads to an increase in PINs transcription in the root, and the optimal auxin concentration for maximum increase differs for each of these genes. Later we showed that transcriptional and posttranscriptional regulation of PIN1 expression by auxin have distinctive features (Omelyanchuk et al., 2016). At the transcriptional level, an increase in PIN1 expression occurs in a wide range of exogenous auxin concentrations, while the PIN1 protein level changes nonlinearly, increasing with raising from low auxin concentration to medium, and then further increase in auxin concentration leads to PIN1 level decreasing.

The major mechanism of auxin-dependent genes regulation occurs through the ARF-Aux/IAA signaling pathway (Ulmasov et al., 1997). When auxin is absent, ARF transcription factors are bound by Aux/IAA co-repressors. Upon entering the cell, auxin interacts with TIR1 receptor, which forms SCF^TIR1^ ubiquitin ligase complex together with other proteins (Dharmasiri et al., 2005; Kepinski, Leyser, 2005). Further, this complex binds to Aux/IAA proteins, regulating their degradation in 26S proteasome (Calderon-Villalobos et al., 2010; Hayashi, 2012). Thus, ARF transcription factors activate or suppress transcription of auxin response genes. In A. thaliana genome, 29 Aux/IAA and 23 ARF genes were found; their expression in different cell types is various, creating sufficient molecular complexity to provide a variety of auxin responses (Remington et al., 2004; Teale et al., 2006). However, it is not known which ARF-Aux/IAA proteins are involved in auxin regulation of PIN expression. It is only known that ARF binding sites were found in promoters of all PINs with bioinformatics methods (Habets, Offringa, 2014).

Reconstruction of the auxin signaling pathway to its PIN transporters is challenging for direct solution by experimental methods. Here, we carried out a meta-analysis of auxininduced transcriptomes in order to obtain a list of genes that significantly change expression together with PINs in response to auxin. A complex approach, including a comparative analysis of these lists and gene networks reconstructed based on those lists, predicted the participants in the ARF-Aux/IAA signaling pathway involved in PIN expression regulation by auxin. Thus, the common signaling pathways for PIN1, PIN3, PIN7 are mediated by combination of ARF4 with IAA12 and IAA18. At the same time, the specific auxin regulation for individual PINs is probably carried out by other proteins of ARF-Aux/IAA signaling pathway. For example, our results showed that ARF10 and IAA32 were present only in the list of genes, which significantly change expression along with PIN4. In addition, we noted the genes that are associated with post-transcriptional regulation of PINs activity in the candidate genes list.

## Materials and methods

Supplementary materials 1–3 are available in the online version of the paper: SUPPLEMENTARY MATERIALS

**Information used in the meta-analysis.** In this study, publicly available data on A. thaliana auxin-induced transcriptomes (microarrays and RNA sequencing) were used. Most of the data were previously presented in (Cherenkov et al., 2018). The summary table of the data has been expanded by the information from (Omelyanchuk et al., 2017). As a result, we took the results of 22 experiments for the meta-analysis. Genes were considered differentially expressed (DEG) if the p-value (according to Benjamini–Hochberg) was less than 0.05. The sets of experiments (Supplementary 1^1^) for each PIN were allocated according to the algorithm we developed (see section “Results. Meta-analysis algorithm”). Work with the summary table and lists of data was carried out using standard methods of Excel (filters, conditional formatting).

**Gene networks reconstruction.** Based on lists of DEGs, gene networks were reconstructed using the String resource (https://string-db.org/) (Szklarczyk et al., 2019). String creates gene networks using user-specified criteria, combining the genes according to the following types of links: experimentally determined (e.g. affinity chromatography), databases (an edge retrieved from the data in databases), textmining (genes found together in publications), co-expression (the same expression patterns of mRNA), neighborhood (calculated based on the proximity of the distance between genes in different genomes), gene fusion (hybrid genes formed in the course of evolution from previously independent genes as a result of chromosomal rearrangements), co-occurrence (presence or absence of linked proteins across species), protein homology. Each link has its own score, calculated through the String algorithms.

## Results

**Meta-analysis algorithm**

Stage 1: data collection. We form a summary table of all publicly available microchip experiments and RNA sequencing data on the topic of interest. In our case, this is information about differentially expressed genes in response to auxin treatment for A. thaliana. The collected data can be geterogenous, for example, our meta-analysis contains data from 22 experiments, containing two samples types (root, whole seedling), three development stages (3-, 5–7-, 10–12 dag seedlings), five time intervals of treatment (0.5–1 h, 2–4 h, 6–8 h, 12–24 h), six types of auxin and its concentrations (0.1; 1; 5; 10 μM IAA; 10 μM NAA; 10 μM IBA).

Stage 2: selection of the experiments appropriate to the task. In the summary table obtained at Stage 1, we find the experiments, in which there was a change in gene expression, for which we are looking for regulators. In accordance with our issue, it is known that A. thaliana has eight PIN transporters. We found PIN1 (in five experiments), PIN3 (in eight experiments), PIN4 (in one experiment) and PIN7 (in six experiments) differentially expressed in these auxin-induced public transcriptomes.

Stage 3: identification of genes that change their expression under auxin influence along with PIN genes. Separately, for each PIN we selected only those DEGs that changed exclusively in experiments where this PIN changes expression, and in other experiments DEG was absent. Thus, we identify genes potentially involved in PIN regulation by auxin. There also may be genes that are direct targets of auxin gradient changes due to PIN proteins activity. For each studied PIN, a table is formed that contains information about activation of suppression of each DEG under auxin treatment. The DEG is marked in the table only if it is differentially expressed along with PIN in at least one experiment.

Stage 4: the formation of DEGs lists that significantly change expression together with PIN. We used the binomial distribution to determine the number of experiments, in which the gene is a DEG along with PIN, to consider this event non-random ( p > 95 %). For each gene list, the significance threshold differs according to amount of experiments, in which a certain PIN is differentially expressed (see Stage 2). In our case, for PIN3 DEG is considered significant if its expression changes occur in three or more experiments, for PIN1 and PIN7 – in two or more experiments. Since PIN4 is differentially expressed only in one experiment, the list of DEGs that change expression along with PIN4 will not vary from Stage 3.

Stage 5: identification of common and specific gene groups. Comparing DEG lists from previous stage with each other we highlight genes found in several lists, i. e. common for PINs, and also mark genes found only in one list, thereby identifying genes that specifically change expression together with a certain PIN.

Stage 6: gene networks reconstruction. Using prepared lists of DEGs from Stage 4, we create gene networks for each PIN and reconstruct interactions between all genes of each list. The connectivity of this network reflects the gene set, for which one of interaction types available in the String database has been found (textminig, co-expression, co-occurrence, etc.).

Stage 7: analysis of gene networks composition. First of all, we pay attention to genes for which links to the genes under study are found in String, paying attention to the type of the interaction. Then from the ontologies list we select biological process that are related to the studied issue. In our study, we chose the auxin-activated signaling pathway.

Using the meta-analysis algorithm described above, we obtained several candidate genes, which regulate PIN expression with a high probability. Next, we describe the results of the reconstruction of auxin signaling pathway to its PIN transporters.

**Meta-analysis of auxin-induced transcriptomes**

**Fig. 1.**
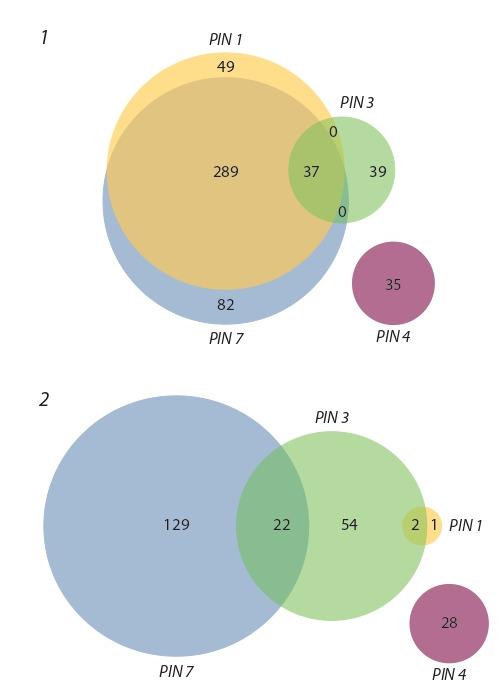
Twelve groups of genes identified in meta-analysis that signif i- cantly change their expression together with PIN1, PIN3, PIN4 and PIN7. 1 – auxin activated genes; 2 – auxin inhibited genes.

Initially, the collected auxin-induced transcriptomes contained more than 20 thousand DEGs that change expression in response to auxin treatment. Among these DEGs, there were four members of PIN family: PIN1, PIN3, PIN4, PIN7. After performing the meta-analysis algorithm described above, we selected four lists of DEGs, jointly changing the expression with PIN1, PIN3, PIN4, PIN7, respectively (Supplementary 2). In total, expression of 531 genes significantly increased and 236 genes decreased their expression jointly with PINs (Fig. 1). Together with PIN1, the expression of 378 genes was significantly altered, of which 375 genes increased the expression level in auxin response similar to PIN1. For the rest of PIN genes, the difference in number of suppressed and activated potential regulators was not so great.

Then, we compared the lists with each other and determined common DEGs for several PINs and specific DEGs to each PIN gene. Twelve groups of genes were obtained: specific auxin-activated genes and specific suppressed genes were found for each PIN, as well as two groups of auxin-activated genes common for (PIN1, PIN3, PIN7) and (PIN1, PIN7); two groups of suppressed genes by auxin, common to (PIN3, PIN7) and (PIN1, PIN3). Activated and suppressed PIN4 potential regulators don’t overlap with those for other PINs. Since among potential regulators of PIN activity there were participants of auxin signaling pathway, we searched for them in the lists (see Supplementary 2) and described to which DEG groups they belong.

**Prediction of auxin-dependent regulators of PIN gene expression**

Since the meta-analysis predicted auxin-dependent regulators of PIN gene expression, we isolated genes for transcriptional and post-transcriptional regulation in DEG lists. We searched for possible transcriptional regulators only among ARF transcription factors and IAA proteins. Possible post-transcriptional regulators have been identified among members of known protein families that affect the PIN protein localization on cell membrane.

**Possible regulators of PIN expression at the transcriptional level**

As a result of meta-analysis, we found that ARF4 and IAA12, IAA18 are the common potential regulators for (PIN1, PIN3, PIN7 ). IAA4 has been identified as a specific regulator for PIN1, while ARF10 and IAA32 presumably mediated auxin response for PIN4. In addition, IAA17 was found in a group of genes that change their expression with PIN1 and PIN7. Interestingly, we didn’t find transcription factors of Aux/IAA family among specific regulators of PIN3 and PIN7, but we did find regulators belonging to other transcription factors families. Therefore, there are obvious differences in ARF-Aux/IAA sets for studied PIN genes, which may also cause differences in dose-dependent regulation of these transporters by auxin.

**Possible regulators of PIN polar localization**

According to the published data, PIN proteins circulate between plasma membrane and cytoplasm in vesicles. This process is regulated by BIG, GN, ARF1 proteins and AGC, PID kinases families, and their functioning is controlled by auxin (Dhonukshe, 2011). Moreover, the polar localization of PIN proteins is also influenced by ABCB1, ABCB19 and ROPGEF protein family (Pan et al., 2015). In the course of data meta-analysis, among DEGs in response to auxin treatment we found a downregulation of BIG4 and ROPGEF11 in gene lists that change expression jointly with PIN7 and PIN4, respectively. An upregulation was noted for WAG2 (member of AGC kinase family) in the group of genes that change their expression along PIN1 and PIN7.

In addition, in our opinion, it is interesting that RGF6/GLV1/ CLEL6 RNA of signal peptide was upregulated in response to auxin in experiments where activity of PIN1 and PIN7 is increased. Another peptide from RGF/GLV/CLEL family, RGF8/GLV6/CLEL2, was increased in experiments where only PIN7 changed expression.

Thus, the formation of auxin response for (PIN1, PIN3, PIN7) group is due to common signaling pathways mediated by ARF4 and IAA12, IAA18. Additionally, there are ARFAux/ IAA specific paths for PIN1 and PIN4. Also among the known auxin-sensitive genes affecting PIN polar localization, we found downregulation of BIG4 and ROPGEF11, which probably contributes to specific responses of PIN7 and PIN4, respectively.

**Reconstruction of gene networks**

**Fig. 2.**
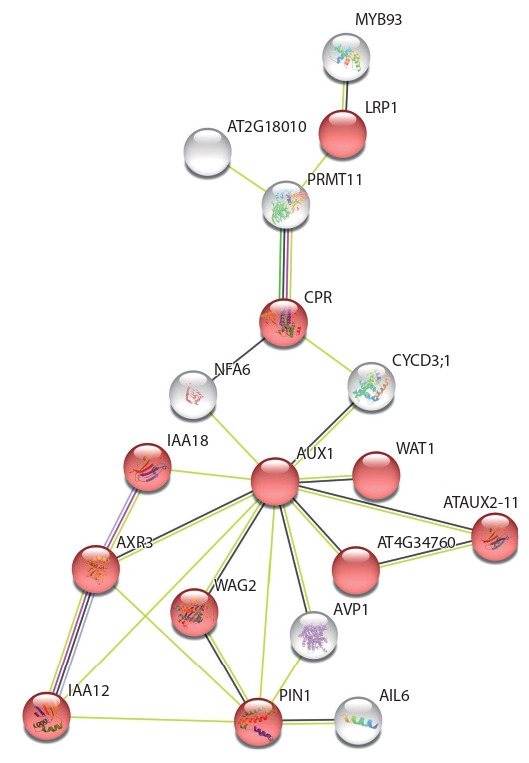
A fragment of the gene network, containing genes associated with PIN1 and genes related to the auxin signaling pathway. Red circles denote genes traditionally related to the auxin signaling pathway. Grey – genes identified in meta-analysis, for which direct or indirect links to PIN1 were found in String. The color of the link reflects what kind of data String used for the creation of interaction. Yellow links are based on textmining; black – on co-expression data, blue – on protein homology, pink – on proteinprotein interactions.

We used the lists of DEGs for each PIN and reconstructed gene networks, which made it possible to evaluate described DEG interaction and, most importantly, how all these DEGs can affect PIN expression activity. As a result, we obtained the connected networks, in which interactions with PIN genes were found, only for PIN1, PIN3 and PIN7. The meta-analysis, from which gene lists for network reconstruction were made, provides significance in itself, so we used a linkage threshold of 0.4. Since we are interested in reconstruction of auxin signaling pathway, we noted only this biological process in String. Notably, most links are formed based on automatic analysis of the articles texts. In the gene network reconstructed based on DEGs that change expression along with PIN1, 12 genes related to the activation of auxin signaling pathway were found (Supplementary 3). At the same time, IAA12, IAA17 (AXR3), WAG2, AUX1 were directly associated with PIN1, the other genes of auxin response were associated with PIN1 indirectly (Fig. 2). It can also be noted that AIL6/PLT3 and AVP1, which are related to the auxin-regulated organ development in Arabidopsis, were directly associated with PIN1 (Krizek, 2011). These genes can be attributed to genes that are direct targets of auxin gradient changes under PIN action. Among these genes, the links between PIN1 and AIL6 and WAG2 were constructed based on co-expression data of RNA sequencing experiments.

Reconstructed gene network for DEGs that alter expression jointly with PIN3 contained eight genes traditionally related to auxin signaling pathway (see Supplementary 3). Direct interactions to PIN3 have been found for AUX1, IAA12 and SAUR9. In the gene network for PIN7, fourteen genes belonged to traditional auxin signaling pathway. At the same time PIN7 directly interacts with IAA12, IAA17 (AXR3), AUX1, LPR1 and WAG2 (see Supplementary 3). In addition, PIN7 had direct links with ABCG33, NFA6, PHOT1, YUC2, YUC6, related to other biological processes controlled by auxin. Reconstruction of gene networks is an additional verification of the fact that regulation of PIN expression by auxin likely occurs with participation of IAA12 and IAA17. It should be noted that the absence of direct connections with PINs for the rest of predicted by meta-analysis ARF-Aux/IAA regulators does not exclude them from the list of candidates for experimental verification in the future.

## Discussion

Phytohormones are actively involved in the processes of plant growth and morphogenesis. The action of auxin in these processes is well studied and it is based on the changes in auxin distribution in tissues (Mroue et al., 2018). Consequently, auxin concentration is a limiting factor in determining cell fate. Proteins-transporters of the PIN family play an important role in the realization of the morphogenetic action of auxin, since they create directed fluxes of this hormone in tissues and, thus, mediate the formation of auxin concentration gradients (Vanneste, Friml, 2009).

An important aspect in the process described above is the presence of positive and negative feedback loops in the mutual regulation of auxin efflux from the cell through PIN functioning and the number of these transporters controlled by auxin. The regulation of auxin-sensitive genes expression is mediated by two proteins families. The first family is ARF transcription factors, which bind to AuxRe site in the promoter of the auxin-sensitive gene and act as an activator or repressor of gene expression (Ulmasov et al., 1997). In some sources, only ARF5-ARF8, ARF19 are supposed to be activators of expression, but there is no experimental confirmation of this (Guilfoyle, Hagen, 2007). The second is the Aux/IAA corepressors, which in the absence of auxin are associated with ARF.

Previously, it was reported, that PIN1–4, PIN7 expression was downregulated in axr3/iaa17 and solitary-root-1(slr-1)/ iaa14 mutants (Vieten et al., 2005) and PIN1 expression is regulated by ARF5 transcription factor (Wenzel et al., 2007), which interacts with IAA12 (Hamann, 2002). In the present work, using computer methods of meta-analysis for genomewide data and gene networks reconstruction, we predicted the details of the auxin signaling pathway to its PIN transporters. The results indicate that there are common mechanisms for PIN1, PIN3, PIN7 and PIN1, PIN7 transcription regulation by auxin, as well as specific mechanisms for PIN expression regulation by auxin. By the common mechanism for PIN1, PIN3, PIN7, we predict the activation of their expression through ARF4-IAA12, ARF4-IAA18, and for PIN1 and PIN7 – additionally through ARF4-IAA17. Specific mechanisms are implemented via ARF4-IAA4 and ARF10-IAA32 for PIN1 and PIN4, respectively. The interactions between these ARFs and IAAs have been experimentally confirmed (Paponov et al., 2008). Recently, it was shown that salinity downregulates PIN expression and leads to stabilization of IAA17 (Liu et al., 2015). Moreover, this type of stress causes a decrease in the size of root apical meristem due to a decline in auxin accumulation, mediated by PIN1, PIN3, PIN7 downregulation. In our data, in auxin-induced transcriptomes, an increase in the expression of PIN1 and PIN7 is accompanied by an increase in IAA17 expression.

For signal peptides of the RGF/GLV/CLEL family, it was previously noted that during gravitropism they change the auxin gradient in the hypocotyl and root (Whitford et al., 2012). At the root, this is due to regulation of PIN2 protein localization by peptides of this family. It was shown that peptides GLV3 and, possibly, GLV6 and GLV9, are secreted from the cortex and endodermis and pass into the outer layers to regulate PIN2 localization. The GLV1 peptide is not expressed in the root, but is present in the hypocotyl, where it also changes the auxin gradient during gravitropism, both during overexpression and loss of function upon mutation (Whitford et al., 2012). According to our data, RGF/GLV/CLEL peptides are involved in the signaling pathway that regulates PIN1 and PIN7 protein localization, and possibly indirectly affect the increase in the expression of these PIN genes. Overexpression or treatment of GLV1 leads to lengthening of the root and its apical meristem due to the fact that the zone of cell division in the root increases, i. e., cells later proceed to differentiation (Fernandez et al., 2013). This transition is also associated with a change in auxin distribution, which is formed by its transporters.

## Conclusion

Thus, created algorithm for the meta-analysis of genome-wide data was applied to finding participants and reconstructing the auxin signaling pathway to its transporters. We were able to reveal that auxin controls PIN1, PIN3, PIN7 expression both through common regulators and specifically, while for PIN4 only specific regulators have been identified. We found published experimental data that partially support our assumptions. As a result of computer research, we have nominated new candidates for experimental verification.

## Conflict of interest

The authors declare no conflict of interest.
