# Supplementary material for: The auxin signaling pathway to its PIN transporters: insights based on a meta-analysis of auxin-induced transcriptomes
Source: Vavilovskii Zhurnal Genet Selektsii. 2021 Feb;25(1):39–45. doi: 10.18699/VJ21.005 (PMC8627910; doi:10.18699/VJ21.005)
Supplement: Supplementary materials 1–3 are available in the online version of the paper: — SUPPLEMENTARY MATERIALS [file VJGB-25-21005-Suppl1.pdf]

ПРИЛОЖЕНИЯ
к статье В.В. Коврижных, З.С. Мустафина, З.З. Багаутдиновой
«Поиск участников сигнального пути ауксина к его транспортерам PIN
на основе метаанализа транскриптомов, индуцированных ауксином»

Приложение 1

Таблица с указанием идентификаторов экспериментов,
используемых для метаанализа для каждого PIN

Название гена	ID экспериментов, в которых ген дифференциально экспрессируется
PIN1	IAA_1h_unpublished<br>GSE97258<br>GDS672_1uM_1h<br>GDS672_1uM_3h<br>GSE42896_6h
PIN3	IAA_1h_unpublished<br>GSE97258<br>GSE35580_iroot<br>GDS672_0.1uM_1h<br>GDS672_1uM_1h<br>GDS672_1uM_3h<br>GSE42896_2h<br>GSE42896_6h
PIN4	GDS672_1uM_1h
PIN7	IAA_1h_unpublished<br>GSE97258<br>GSE42007_8h<br>GDS672_1uM_1h<br>GDS672_1uM_3h<br>GSE42896_6h

Приложение 2

PIN1		PIN3		PIN4		PIN7	
Активация	Подавление	Активация	Подавление	Активация	Подавление	Активация	Подавление
AT1G30240	AT2G16980	AT1G70940	AT4G33150	AT3G50340	AT2G26400	AT1G51950	AT1G23080
AT1G31810	AT2G30520	AT1G51950	AT4G35030	AT3G58120	AT3G45730	AT1G30240	AT1G30260
AT1G33590	AT2G30930	AT5G60450	AT4G35060	AT4G02290	AT3G49620	AT1G31810	AT1G31770
AT1G48920	AT2G19110	AT4G31910	AT4G36380	AT4G25420	AT4G23140	AT1G33590	AT2G39730
AT1G51950		AT1G33590	AT4G39030	AT4G32290	AT4G26460	AT1G48920	AT2G41660
AT1G58170		AT1G57560	AT4G39940	AT5G06930	AT4G33050	AT1G58380	AT2G47600
AT1G58380		AT2G23180	AT5G01520	AT5G10520	AT4G34630	AT2G23180	AT3G44320
AT2G23180		AT2G26480	AT5G07580	AT5G10930	AT5G11290	AT2G33210	AT3G45700
AT2G33210		AT3G50660	AT5G09440	AT5G15580	AT5G17220	AT2G40940	AT3G45710
AT2G41510		AT3G54000	AT5G10430	AT5G22310	AT5G24210	AT2G41510	AT3G45780
AT3G44750		AT3G60650	AT5G15830	AT5G39860	AT5G58120	AT3G44610	AT3G46130
AT3G47780		AT3G63240	AT5G22630	AT5G45670	AT1G22150	AT3G44750	AT3G46490
AT3G48970		AT3G63440	AT5G23020	AT5G50335	AT1G31950	AT3G49010	AT3G46700
AT3G49010		AT4G08040	AT5G25810	AT5G51720	AT1G52240	AT3G49960	AT3G49120
AT3G49350		AT4G09890	AT5G27350	AT5G54190	AT1G56510	AT3G49990	AT3G49780
AT3G49360		AT4G11280	AT5G40450	AT1G14550	AT1G69720	AT3G50890	AT3G51240
AT3G49960		AT4G13130	AT5G44020	AT1G18400	AT1G72930	AT3G50930	AT3G52480
AT3G49990		AT4G13235	AT5G49360	AT1G27210	AT1G73805	AT3G51800	AT3G52840
AT3G50400		AT4G17615	AT5G49730	AT1G29440	AT2G03760	AT3G52140	AT3G53100
AT3G50890		AT4G17785	AT5G57150	AT1G29450	AT2G04040	AT3G52710	AT3G53420
AT3G50930		AT4G22780	AT5G59090	AT1G30100	AT2G14560	AT3G54260	AT3G53980
AT3G51800		AT4G26540	AT5G61420	AT1G65920	AT2G17040	AT3G54790	AT3G54590
AT3G52140		AT4G27290	AT5G61480	AT1G68650	AT2G19850	AT3G54950	AT3G54720
AT3G52710		AT4G30410	AT5G65210	AT1G71870	AT2G21210	AT3G55060	AT3G54820
AT3G53540		AT4G30420	AT1G25230	AT2G01200	AT2G36770	AT3G55690	AT3G54960
AT3G54260		AT4G34710	AT1G31770	AT2G01420	AT2G38210	AT3G56070	AT3G56000
AT3G54790		AT4G35200	AT3G46700	AT2G14920	AT2G42850	AT3G56150	AT3G56940
AT3G54950		AT4G35210	AT3G53420	AT2G22420	AT3G20400	AT3G57150	AT3G58990
AT3G55090		AT4G36110	AT3G59710	AT2G22810	AT2G01420	AT3G57550	AT3G59690
AT3G55690		AT4G36360	AT3G61890	AT2G28350		AT3G57660	AT3G59710
AT3G56070		AT4G37290	AT3G62040	AT2G36885		AT3G58660	AT3G61630
AT3G56150		AT4G37370	AT4G00910	AT2G40610		AT3G59820	AT3G62040
AT3G57150		AT4G37590	AT4G04840	AT2G43870		AT3G60220	AT3G62680
AT3G57660		AT4G37890	AT4G18010	AT3G02170		AT3G60640	AT3G63470
AT3G58660		AT4G38850	AT4G21850	AT3G03830		AT3G60840	AT4G00780
AT3G59820		AT5G03550	AT4G30460	AT3G20865		AT3G62310	AT4G00910
AT3G60550		AT5G03670	AT4G31730	AT3G26960		AT3G63240	AT4G01150
AT3G60640		AT5G05160	AT1G05260	AT2G01420		AT4G00940	AT4G01850
AT3G60840		AT5G06080	AT1G13110			AT4G01630	AT4G02270
AT3G62310		AT5G06860	AT1G15210			AT4G01920	AT4G02850
AT3G63240		AT5G09800	AT1G17190			AT4G02450	AT4G04610
AT4G00940		AT5G19530	AT1G20620			AT4G02540	AT4G04830
AT4G01870		AT5G27760	AT1G21440			AT4G02800	AT4G04840
AT4G01920		AT5G51380	AT1G22530			AT4G05190	AT4G08290
AT4G02450		AT5G51670	AT1G27030			AT4G07410	AT4G08300
AT4G07410		AT5G53290	AT1G32640			AT4G08770	AT4G08410
AT4G08780		AT5G54910	AT1G33240			AT4G08780	AT4G10500
AT4G09570		AT5G56030	AT1G67110			AT4G09570	AT4G11310
AT4G10480		AT5G58580	AT1G68440			AT4G10480	AT4G12310
AT4G11230		AT5G61010	AT1G69080			AT4G11160	AT4G13260
AT4G11420		AT5G64250	AT1G72510			AT4G11230	AT4G13440
AT4G12110		AT5G66460	AT1G74090			AT4G11420	AT4G14040
AT4G12480		AT5G67430	AT1G74840			AT4G12110	AT4G14130
AT4G12600		AT1G04550	AT1G74940			AT4G12480	AT4G15560
AT4G12620		AT1G02400	AT1G75750			AT4G12600	AT4G15920
AT4G13235		AT1G02900	AT1G76890			AT4G12620	AT4G16563
AT4G13850		AT1G03820	AT1G78090			AT4G13235	AT4G17340
AT4G14365		AT1G13195	AT2G15050			AT4G13710	AT4G18510
AT4G16515		AT1G18100	AT2G21560			AT4G13850	AT4G18640
AT4G16630		AT1G18670	AT2G24580			AT4G14350	AT4G19420
AT4G18970		AT1G19230	AT2G30520			AT4G14365	AT4G21850
AT4G20320		AT1G21390	AT2G30930			AT4G14390	AT4G23100
AT4G20830		AT1G21980	AT2G36950			AT4G15780	AT4G23400
AT4G20980		AT1G22880	AT2G37180			AT4G16515	AT4G23690
AT4G21200		AT1G28370	AT2G38760			AT4G16630	AT4G25820
AT4G22530		AT1G28400	AT2G39705			AT4G18910	AT4G27030
AT4G22560		AT1G29430	AT2G44380			AT4G18970	AT4G27440
AT4G22610		AT1G33420	AT2G44500			AT4G20320	AT4G28410
AT4G22710		AT1G33970	AT2G46650			AT4G20830	AT4G30460
AT4G22780		AT1G34670	AT2G48030			AT4G20980	AT4G31500
AT4G23750		AT1G51170	AT3G02850			AT4G22470	AT4G32410

PIN1		PIN3		PIN4		PIN7	
Активация	Подавление	Активация	Подавление	Активация	Подавление	Активация	Подавление
AT4G24130		AT1G59740	AT3G13730			AT4G22530	AT4G33420
AT4G24830		AT1G64390	AT3G19030			AT4G22560	AT4G33790
AT4G24960		AT1G70550	AT3G20015			AT4G22610	AT4G35060
AT4G25340		AT1G70920	AT3G21770			AT4G22620	AT4G35090
AT4G25630		AT1G70940	AT3G23800			AT4G22710	AT4G35380
AT4G25730		AT1G71400	AT1G70940			AT4G22780	AT4G37530
AT4G26230		AT1G73590				AT4G23750	AT4G38060
AT4G26540		AT1G74660				AT4G24830	AT4G38620
AT4G26600		AT2G23450				AT4G24970	AT4G39030
AT4G27010		AT2G25690				AT4G25340	AT4G39350
AT4G27280		AT2G30040				AT4G25630	AT4G39660
AT4G28450		AT2G36220				AT4G25700	AT4G39940
AT4G29510		AT2G38120				AT4G25730	AT4G39950
AT4G30150		AT2G39700				AT4G26230	AT4G40090
AT4G30990		AT2G41380				AT4G26540	AT5G01210
AT4G31910		AT2G41820				AT4G26600	AT5G01740
AT4G32520		AT2G42430				AT4G27010	AT5G01800
AT4G32920		AT2G42440				AT4G27280	AT5G02230
AT4G33180		AT2G43290				AT4G28160	AT5G03240
AT4G34160		AT2G47990				AT4G28450	AT5G03380
AT4G34740		AT3G06370				AT4G29510	AT5G03555
AT4G34760		AT3G06490				AT4G30140	AT5G04960
AT4G35200		AT3G11150				AT4G30150	AT5G05170
AT4G35620		AT3G13380				AT4G30850	AT5G06320
AT4G35720		AT3G13790				AT4G30990	AT5G06640
AT4G36020		AT3G13810				AT4G32520	AT5G07460
AT4G36360		AT3G18560				AT4G32920	AT5G07580
AT4G36610		AT3G20940				AT4G33180	AT5G09440
AT4G36680		AT3G21700				AT4G34160	AT5G10430
AT4G37090		AT3G22660				AT4G34740	AT5G10470
AT4G37770		AT3G24080				AT4G34760	AT5G10770
AT4G37910		AT3G24240				AT4G35200	AT5G12250
AT4G38900		AT3G25290				AT4G35620	AT5G12870
AT5G02490		AT3G26760				AT4G35720	AT5G13630
AT5G02500		AT3G28420				AT4G36020	AT5G14120
AT5G02540		AT3G28850				AT4G36360	AT5G15180
AT5G02870						AT4G36680	AT5G15850
AT5G03550						AT4G37090	AT5G16010
AT5G03640						AT4G37770	AT5G18130
AT5G03740						AT4G37900	AT5G22630
AT5G04870						AT4G37910	AT5G23020
AT5G04980						AT5G02490	AT5G23220
AT5G05160						AT5G02500	AT5G23660
AT5G06080						AT5G02540	AT5G24120
AT5G08570						AT5G02870	AT5G25240
AT5G09510						AT5G03550	AT5G25620
AT5G10510						AT5G03740	AT5G35190
AT5G11240						AT5G04870	AT5G35940
AT5G12050						AT5G05160	AT5G38550
AT5G12330						AT5G05180	AT5G38980
AT5G12940						AT5G06080	AT5G40450
AT5G13580						AT5G08570	AT5G40890
AT5G14050						AT5G09510	AT5G41380
AT5G14130						AT5G10010	AT5G42180
AT5G14520						AT5G10510	AT5G42680
AT5G14610						AT5G11240	AT5G44190
AT5G14690						AT5G11300	AT5G45650
AT5G14730						AT5G12050	AT5G47560
AT5G16130						AT5G12330	AT5G47720
AT5G17160						AT5G12940	AT5G47950
AT5G18470						AT5G14050	AT5G48000
AT5G19440						AT5G14520	AT5G48110
AT5G19530						AT5G14610	AT5G49270
AT5G19820						AT5G14650	AT5G49730
AT5G20160						AT5G14690	AT5G49900
AT5G20270						AT5G15160	AT5G51970
AT5G20290						AT5G16130	AT5G52060
AT5G21120						AT5G17160	AT5G54160
AT5G21160						AT5G17340	AT5G54770
AT5G22650						AT5G18470	AT5G57770
AT5G22740						AT5G18550	AT5G59090
AT5G23270						AT5G18560	AT5G61420
AT5G25190						AT5G19530	AT5G61590
AT5G25780						AT5G19820	AT5G62360

PIN1		PIN3		PIN4		PIN7	
Активация	Подавление	Активация	Подавление	Активация	Подавление	Активация	Подавление
AT5G26340						AT5G20160	AT5G63600
AT5G26860						AT5G20270	AT5G64570
AT5G27330						AT5G20290	AT5G64740
AT5G36960						AT5G21160	AT5G65020
AT5G39050						AT5G22430	AT5G65210
AT5G39850						AT5G22500	AT5G65390
AT5G40480						AT5G22650	AT5G66390
AT5G41400						AT5G22740	
AT5G41520						AT5G23270	
AT5G43700						AT5G25780	
AT5G46360						AT5G26340	
AT5G47060						AT5G26860	
AT5G47440						AT5G27330	
AT5G48180						AT5G36960	
AT5G49160						AT5G39850	
AT5G49480						AT5G40210	
AT5G49680						AT5G40480	
AT5G51200						AT5G40780	
AT5G51670						AT5G41520	
AT5G51830						AT5G43830	
AT5G52050						AT5G46360	
AT5G52310						AT5G47060	
AT5G52470						AT5G47440	
AT5G52810						AT5G48180	
AT5G52910						AT5G49160	
AT5G53320						AT5G49480	
AT5G53760						AT5G49680	
AT5G54130						AT5G50180	
AT5G56000						AT5G51200	
AT5G56320						AT5G51670	
AT5G57340						AT5G51830	
AT5G58620						AT5G52050	
AT5G59010						AT5G52470	
AT5G60350						AT5G52810	
AT5G60450						AT5G52870	
AT5G60930						AT5G52890	
AT5G61020						AT5G52910	
AT5G61030						AT5G53320	
AT5G61890						AT5G53760	
AT5G62190						AT5G54130	
AT5G64420						AT5G56000	
AT5G64660						AT5G56320	
AT5G65510						AT5G56740	
AT5G67430						AT5G57070	
AT5G67630						AT5G57100	
AT1G01120						AT5G58620	
AT1G02740						AT5G58750	
AT1G02900						AT5G59010	
AT1G03110						AT5G60350	
AT1G03530						AT5G60450	
AT1G03820						AT5G60930	
AT1G04250						AT5G61020	
AT1G04270						AT5G61030	
AT1G04550						AT5G61890	
AT1G05680						AT5G62190	
AT1G05710						AT5G62260	
AT1G06670						AT5G63810	
AT1G06720						AT5G64420	
AT1G07690						AT5G64660	
AT1G08280						AT5G65510	
AT1G09350						AT5G65640	
AT1G11000						AT5G65890	
AT1G12080						AT5G67130	
AT1G14320						AT5G67430	
AT1G14610						AT5G67630	
AT1G15440						AT1G04250	
AT1G15690						AT1G04550	
AT1G17500						AT1G01120	
AT1G18670						AT1G02740	
AT1G20700						AT1G02900	
AT1G22490						AT1G03110	
AT1G22880						AT1G03530	
AT1G23340						AT1G03820	
AT1G28370						AT1G04030	

PIN1		PIN3		PIN4		PIN7	
Активация	Подавление	Активация	Подавление	Активация	Подавление	Активация	Подавление
AT1G28400						AT1G04270	
AT1G29250						AT1G05680	
AT1G29900						AT1G06670	
AT1G29940						AT1G06720	
AT1G30820						AT1G07690	
AT1G33140						AT1G08280	
AT1G34670						AT1G09350	
AT1G36940						AT1G10380	
AT1G37130						AT1G11000	
AT1G43170						AT1G12080	
AT1G48330						AT1G13380	
AT1G49310						AT1G14320	
AT1G49760						AT1G14610	
AT1G49870						AT1G15440	
AT1G50110						AT1G15660	
AT1G50570						AT1G15690	
AT1G50920						AT1G17500	
AT1G51420						AT1G18670	
AT1G53380						AT1G19200	
AT1G56110						AT1G20700	
AT1G58340						AT1G22030	
AT1G59700						AT1G22490	
AT1G59740						AT1G22880	
AT1G60730						AT1G23080	
AT1G60750						AT1G23340	
AT1G61580						AT1G28370	
AT1G61730						AT1G28400	
AT1G63810						AT1G29250	
AT1G64390						AT1G29900	
AT1G64400						AT1G29940	
AT1G64790						AT1G30820	
AT1G64880						AT1G33140	
AT1G64900						AT1G34670	
AT1G67120						AT1G36940	
AT1G69070						AT1G37130	
AT1G70550						AT1G43170	
AT1G71400						AT1G48330	
AT1G72230						AT1G49760	
AT1G72680						AT1G49870	
AT1G73590						AT1G50110	
AT1G74450						AT1G50570	
AT1G74460						AT1G50660	
AT1G74560						AT1G50920	
AT1G74790						AT1G53380	
AT1G75500						AT1G55580	
AT1G76540						AT1G55610	
AT1G78120						AT1G56110	
AT1G79150						AT1G58340	
AT1G79450						AT1G59740	
AT1G79470						AT1G60730	
AT1G80410						AT1G61580	
AT2G01250						AT1G61730	
AT2G01430						AT1G63810	
AT2G03090						AT1G64390	
AT2G03730						AT1G64400	
AT2G04030						AT1G64610	
AT2G04100						AT1G64790	
AT2G15480						AT1G64880	
AT2G15490						AT1G67120	
AT2G16500						AT1G69070	
AT2G18010						AT1G70550	
AT2G18900						AT1G71400	
AT2G20450						AT1G72230	
AT2G21030						AT1G73590	
AT2G23450						AT1G74160	
AT2G25520						AT1G74450	
AT2G25790						AT1G74560	
AT2G26250						AT1G74790	
AT2G26290						AT1G75500	
AT2G27000						AT1G75520	
AT2G27010						AT1G76160	
AT2G27840						AT1G76540	
AT2G28000						AT1G78120	
AT2G29420						AT1G78530	

PIN1		PIN3		PIN4		PIN7	
Активация	Подавление	Активация	Подавление	Активация	Подавление	Активация	Подавление
AT2G29440						AT1G79150	
AT2G29460						AT1G79450	
AT2G29490						AT1G79470	
AT2G30140						AT1G80410	
AT2G31660						AT2G01250	
AT2G32070						AT2G01430	
AT2G32560						AT2G03090	
AT2G34660						AT2G03730	
AT2G36220						AT2G03830	
AT2G37190						AT2G04030	
AT2G37210						AT2G04100	
AT2G37270						AT2G16500	
AT2G38120						AT2G18010	
AT2G39220						AT2G18900	
AT2G39230						AT2G19520	
AT2G39350						AT2G20450	
AT2G39360						AT2G21030	
AT2G39380						AT2G23450	
AT2G39420						AT2G23530	
AT2G39700						AT2G25520	
AT2G40360						AT2G26250	
AT2G40430						AT2G27000	
AT2G43290						AT2G27010	
AT2G43500						AT2G28000	
AT2G43590						AT2G28660	
AT2G43650						AT2G29440	
AT2G45970						AT2G29460	
AT2G46950						AT2G31660	
AT3G01160						AT2G32070	
AT3G03130						AT2G32560	
AT3G03660						AT2G34555	
AT3G03920						AT2G34920	
AT3G04840						AT2G36220	
AT3G05060						AT2G37190	
AT3G05590						AT2G37210	
AT3G06490						AT2G37270	
AT3G06530						AT2G37280	
AT3G07050						AT2G38010	
AT3G07770						AT2G38120	
AT3G08030						AT2G38480	
AT3G09980						AT2G39220	
AT3G10050						AT2G39230	
AT3G10600						AT2G39350	
AT3G10650						AT2G39360	
AT3G11964						AT2G39380	
AT3G12270						AT2G39420	
AT3G12670						AT2G39700	
AT3G12700						AT2G39870	
AT3G12860						AT2G40360	
AT3G13380						AT2G40430	
AT3G13470						AT2G43290	
AT3G13790						AT2G43500	
AT3G14370						AT2G43590	
AT3G15000						AT2G43650	
AT3G15250						AT2G45970	
AT3G16780						AT2G46740	
AT3G18130						AT3G01160	
AT3G18560						AT3G03130	
AT3G20050						AT3G03660	
AT3G20940						AT3G03920	
AT3G21420						AT3G04570	
AT3G21700						AT3G04840	
AT3G22600						AT3G05060	
AT3G22620						AT3G05590	
AT3G23620						AT3G05830	
AT3G23830						AT3G06490	
AT3G23890						AT3G06530	
AT3G23990						AT3G07050	
AT3G24240						AT3G07770	
AT3G25100						AT3G08030	
AT3G25230						AT3G09760	
AT3G25640						AT3G09980	
AT3G26470						AT3G10050	
AT3G26750						AT3G10600	
AT3G26850						AT3G10650	
AT1G73590						AT3G11964	

PIN1		PIN3		PIN4		PIN7	
Активация	Подавление	Активация	Подавление	Активация	Подавление	Активация	Подавление
						AT3G12270<br>AT3G12670<br>AT3G12700<br>AT3G12860<br>AT3G13380<br>AT3G13470<br>AT3G13790<br>AT3G14370<br>AT3G14610<br>AT3G15000<br>AT3G15250<br>AT3G16310<br>AT3G16780<br>AT3G18130<br>AT3G18400<br>AT3G18560<br>AT3G20050<br>AT3G20830<br>AT3G20940<br>AT3G21420<br>AT3G21700<br>AT3G22600<br>AT3G22620<br>AT3G23620<br>AT3G23830<br>AT3G23890<br>AT3G23990<br>AT3G24240<br>AT3G24810<br>AT3G25100<br>AT3G25230<br>AT3G26750<br>AT3G26850<br>AT3G30380<br>AT1G23080	

Приложение 3

Полные версии реконструированных генных сетей для PIN1, PIN3, PIN7 на основе полученных в метаанализе списков ДЭГ

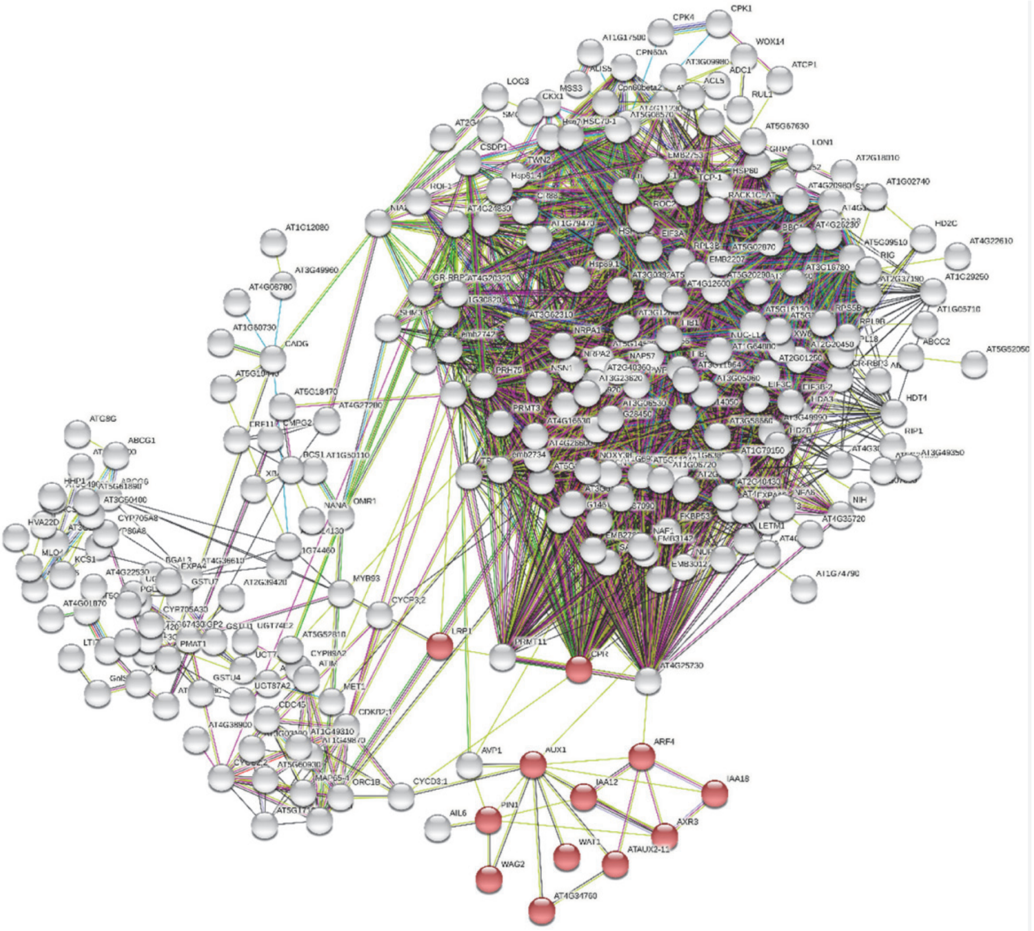

Рис. 1. Генная сеть ДЭГ, изменяющих экспрессию вместе с PIN1.

Красными кругами обозначены гены, традиционно относящиеся к сигнальному пути ауксина, серыми – гены, выявленные в метаанализе, для которых в String были найдены прямые или опосредованные связи к PIN1. Цвет связи отражает, на основе каких данных из String построено взаимодействие, и соответствует используемому в ресурсе.

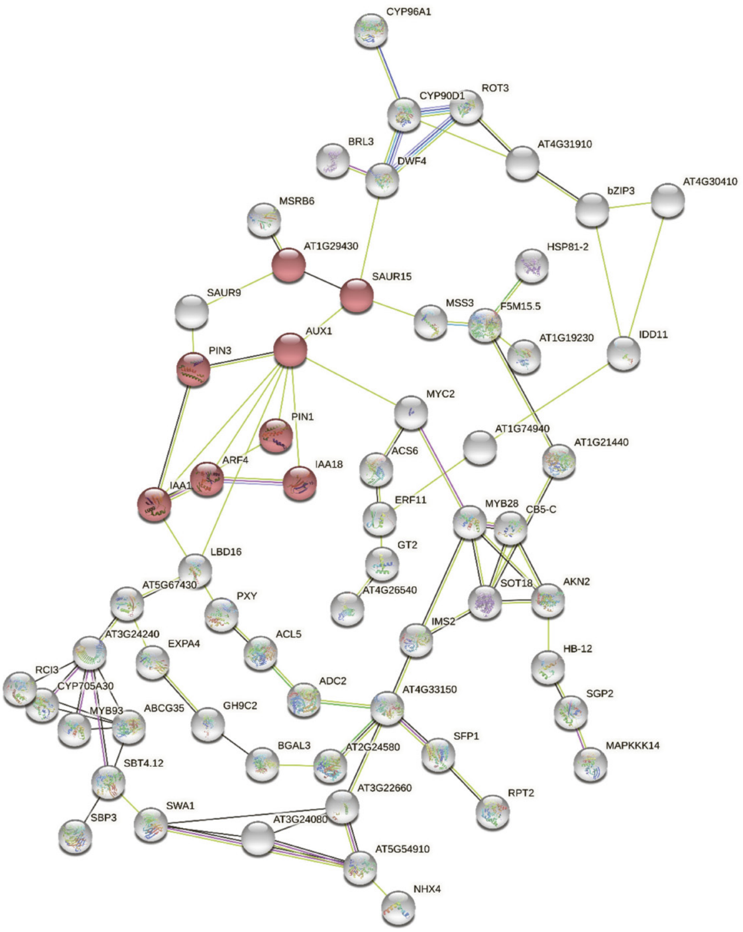

Рис. 2. Генная сеть ДЭГ, изменяющих экспрессию вместе с PIN3.

Красными кругами обозначены гены, традиционно относящиеся к сигнальному пути ауксина, серыми – гены, выявленные в метаанализе, для которых в String были найдены прямые или опосредованные связи к PIN3. Цвет связи отражает, на основе каких данных из String построено взаимодействие.

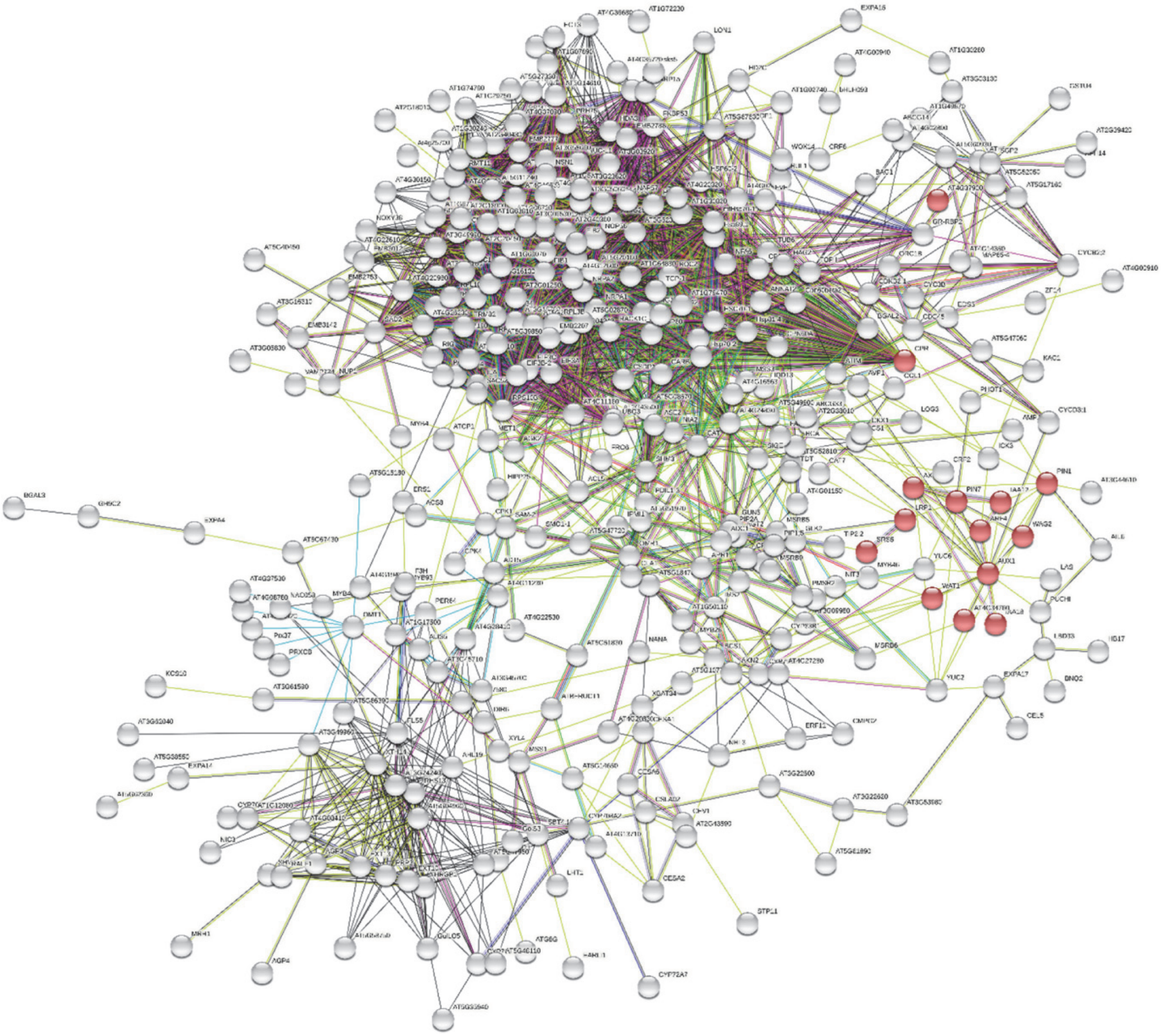

Рис. 3. Генная сеть ДЭГ, изменяющих экспрессию вместе с PIN7.

Красными кругами обозначены гены, традиционно относящиеся к сигнальному пути ауксина, серыми – гены, выявленные в метаанализе, для которых в String были найдены прямые или опосредованные связи к PIN7. Цвет связи отражает, на основе каких данных из String построено взаимодействие.
